# Enhanced Detection of αGal Using a Novel Monoclonal IgG1 Antibody: Comparative Evaluation with IgM Antibody [Clone M86]

**DOI:** 10.3390/jpm15110558

**Published:** 2025-11-17

**Authors:** Rosa Di Liddo, Filippo Naso, Alessandro Gandaglia, Giulio Sturaro, Michele Spina, Robert J. Melder

**Affiliations:** 1 Department of Pharmaceutical and Pharmacological Sciences, University of Padova, 35131 Padova, Italy; 2 Biocompatibility Innovation S.r.l., 35042 Este, Italy; a.gandaglia@bci-biocompatibility.com (A.G.);; 3 Department of Biomedical Sciences, University of Padova, 35122 Padova, Italy; michele.spina@unipd.it; 4 Mountain Hawk Consulting LLC, Glen Allen, VA 23059, USA

**Keywords:** αGal, monoclonal antibody, IgM-M86, bioprosthesis, regulatory agencies

## Abstract

**Introduction.** Over the past two decades, the αGal (Galα1–3Galβ1–4GlcNAc–R) epitope, a carbohydrate found in many non-primate mammals, has gained significant relevance in medicine due to its association with an increasing number of allergic reactions to animal-derived foods, drugs, and medical devices. Due to a mutated gene coding for α1,3-galactosyltransferase (α1–3GT), humans lack αGal and, therefore, naturally produce anti-α-Gal antibodies (IgM, IgA, and IgG), especially in the context of a xenotransplantation, which can lead to extreme immunological reactivity, including hyperacute rejection of the transplant. Recently, these uncontrollable immune reactions have driven demand for more accurate procedures to better detect αGal in animal-derived foods or bioprosthetics. The currently most widely used α-Gal-specific monoclonal antibody is an IgM antibody (clone M86), developed in *Ggta1* KO mice and isolated from hybridoma tissue culture. As the IgM isotype has limited purification properties, specificity, and sensitivity, we aimed to produce a novel IgG antibody with high affinity and extensive applicability. **Methods.** An experimental murine IgG1 anti-αGal antibody (IgG-αGalomab) was developed by immunization of *Ggta1* knockout (KO) mice, and its affinity was evaluated using ELISA, Western blot, flow cytometry, and immunohistochemistry/immunofluorescence. **Results.** Compared to IgM-M86, IgG-αGalomab demonstrated ~1200-fold higher binding potency and lower cross-reactivity with competitive molecules, i.e., bovine serum albumin, galactobiose, and lactose. Unlike IgM-M86, IgG-αGalomab showed an increasing affinity over time in the binding tests performed on xenogeneic tissues. Notably, high-affinity for αGal was detected by Western blot at high dilution [1:200,000] of IgG-αGalomab compared to IgM-M86 [1:1000]. By flow cytometry, specificity and dose-dependent response were confirmed using in vitro cultures of porcine and human fibroblasts. Finally, in immunofluorescence and immunohistochemistry analysis, αGal was demonstrated to be detectable by IgG-αGalomab at a dilution of [1:1000], while IgM-M86 was demonstrated to be detectable at [1:100]. **Conclusions.** Altogether, our newly developed antibody showed high sensitivity and specificity for α-Gal in various applications. Based on its potential binding capacity, IgG-αGalomab could have important applications in precision medicine for predicting, treating, and preventing immune-mediated phenomena of patients in different medical areas.

## 1. Introduction

**Figure 1:**
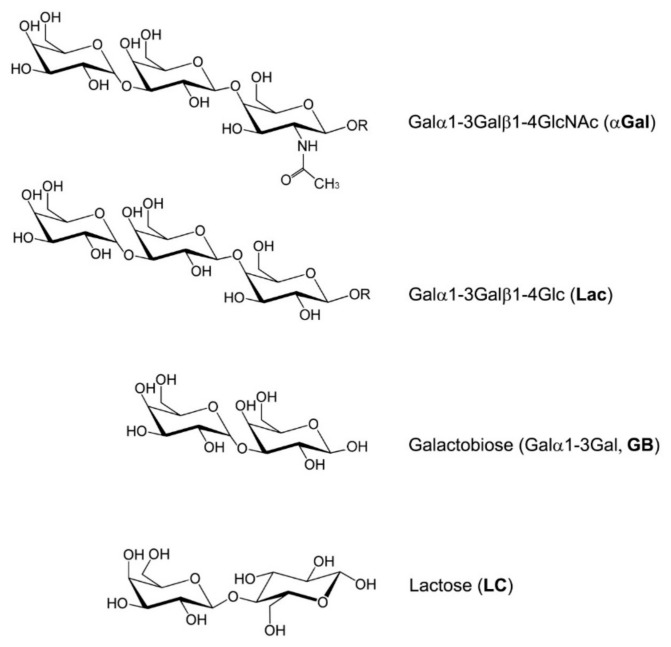
Acetylated (α-Gal, immunogenic) and non-acetylated (Galactobiose, GB, non-immunogenic) variants of galactose α-1,3-galactose. In the present study, Lac, GB, and lactose (LC) were used as competitors to evaluate and compare the binding affinities of IgG-αGalomab or IgM-M86 antibody to the αGal antigen.

The αGal antigen (Galα1-3Galβ1-4GlcNAc-R) is an oligosaccharide present in the cell membranes of most mammals, except primates and humans (Figure 1) [1]. This different expression in humans and higher primates is due to a loss-of-function mutation in the gene encoding the enzyme alpha-1,3-galactosyltransferase (*Ggta1*).

αGal immunogenicity is known to cause the production of IgM (normal serum), IgA (saliva, vaginal washings, bile, colostrum, milk, and normal serum), and IgG subclasses of immunoglobulins [2]. Since it is found in the lipopolysaccharide and capsular polysaccharides of some Gram-negative bacteria in the human gastrointestinal tract, αGal is reported to stimulate the immune system over time, contributing to the development of circulating anti-Gal antibodies (~1–3% of circulating IgG) [3].

Besides immunogenicity, which refers to the ability of the antigen to trigger a protective immune response, αGal is characterized by allergenicity, that is, the ability of a substance to induce an abnormal hyperimmune reaction causing a physiological function disorder [4].

In some individuals, the natural immune response to αGal can be skewed towards IgE production, leading to allergic reactions and the development of Alpha-Gal Syndrome (AGS), a condition that occurs immediately when αGal is administered parenterally, whereas it appears after several hours when it is transmitted via the oral route [5].

In clinical practice, the production of bioprosthetic systems (i.e., pericardial patches, ligaments, tendons, and heart valves) using animal-derived tissues implies a high risk of triggering an αGal-mediated response. Correlated with degenerative occurrences, bioprosthetic devices exhibit limited functionality over time and a high rate of bioprosthesis reintervention [6,7,8,9]. Consequently, αGal allergies may arise as early as a few hours after implantation due to the presence of anti-Gal antibodies in recipients.

Sensitization to αGal usually occurs when the antigen is transmitted into the human bloodstream through a tick bite. Subsequent exposure to mammalian red meat, dairy products, or drugs of mammalian origin (such as gelatin, lactose, antivenoms, or heparins) can promote allergic reactions [10].

Currently, regulatory agencies such as the FDA, EMA, and EU Regulations on Medical Devices (MDR) do not require a general obligation to disclose the αGal content in animal-derived bioprostheses or drugs intended for commercialization, despite the recent incorporation of glycan epitopes into the WHO/IUIS Allergen database [4]. Recently, considerable attention has been focused on the αGal expression in implanted materials to better understand the prevalence and risks associated with antigen exposure [5,11]. A key example of the growing recognition of the medical challenges and health risks associated with αGal is the FDA’s 2020 approval of Galsafe, a genetically engineered pig lacking the αGal antigen [12]. This genetic modification in a line of domestic pigs is specifically engineered to help overcome food and medical challenges associated with the presence of this antigen.

Until now, the qualitative and/or quantitative evaluation of this antigen in biological matrices has been performed using a single commercially available mouse anti-αGal IgM antibody (M86 clone) [13,14], which was developed in *Ggta1* KO mice and isolated from hybridoma tissue culture. However, it shows some limitations, such as its pentameric structure and high molecular weight, which are not particularly suitable for quantitative assays. Alternative approaches for detecting and quantifying the αGal antigen have also been explored and reported in the literature. Notably, Cunningham et al. engineered a single-chain antibody fragment derived from a chicken, specifically targeting the αGal antigen, which has been validated to a limited extent for use in the ELISA procedure [15]. In addition, Kreft L. and co-workers described the synthesis and validation of a novel IgG1 monoclonal antibody against αGal with a high affinity and specificity [16].

In this study, an experimental murine monoclonal IgG1 antibody, designated as IgG-αGalomab, has been produced against the αGal epitope and tested in both quantitative and qualitative assays, including ELISA, Western blot, flow cytometry, and immunofluorescence/immunohistochemistry. For validation, anti-αGal monoclonal antibody IgM-M86 has been used as a reference.

## 2. Materials and Methods

### 2.1. Reagents

All products were purchased from Sigma-Aldrich/Merck (Burlington, MA, USA) and Dextra Laboratories (Reading, UK) except those otherwise specified; (ALX-801-090-1) α-Gal Epitope (Galα1-3Galβ1-4GlcNAc-R) monoclonal antibody (M86) (starting recommended dilution [1:5]) was provided by Enzo Life Sciences (Farmingdale, NY, USA); the IgG-αGalomab antibody was developed and produced by Biocompatibility Innovation S.r.l., (Este, Padua, Italy) in collaboration with GenScript Biotech (Rijswijk, The Netherlands).

### 2.2. Animal Ethics Statement

All animal experimental procedures were performed in accordance with relevant regulations and guidelines, such as the European Convention for Animal Care and Use of Laboratory Animals. *Ggta1* knockout (KO) and wild-type (WT) mice were kept under specific pathogen-free conditions.

### 2.3. Production of IgG-αGalomab: Immunization Protocol and Hybridoma Generation

Starting from a *Ggta1* KO mouse model, which is a validated system for the experimental study of allogeneic bioprostheses for human use [17,18,19], the production of a hybridoma was performed as previously described by Milstein and Kohler [20]. Briefly, mice were immunized subcutaneously and intraperitoneally with 50 µg αGal/BSA (Dextra Laboratories Ltd., Thames Valley Science Park, Reading 9LH, UK), three times, with 3-week intervals between each administration. An aliquot of 10 µL of serum from each animal was then used to assess the level of anti-αGal IgG antibodies by the ELISA technique. When an appropriate antibody titer was detected, the animals were sacrificed, and B lymphocytes (approximately 10^8^ cells) were isolated from their spleens and fused with a mouse myeloma-derived cell line (approximately 10^7^ cells) to generate hybridomas. Cell suspensions containing fused cells were seeded into 96-well culture plates and cultured in selective HAT (hypoxanthine–aminopterin–thymidine) medium (Thermo Fisher Scientific, Waltham, MA, USA), in a humidified atmosphere containing 5% CO_2_ at 37 °C. The samples were monitored for the hybridoma growth over 10–14 days. Culture supernatants showing visible colonies were screened by indirect ELISA for the presence of anti-αGal antibodies. Positive samples were identified and expanded into 24-well plates for a secondary screening of antibody production and specificity.

Hybridomas showing the strongest and most specific signal were subjected to at least two additional rounds of limiting dilution cloning to ensure monoclonality and genetic stability. Each subcloning step involved single-cell seeding per well under identical selection conditions, followed by rescreening of the obtained supernatants. Five clones were recloned, and ten new cell lines (two for each original clone—see Table 1) were generated.

Due to application-oriented evaluations based on αGal detection in biological matrices (i.e., bioprosthetic heart valves) commonly used in clinical practice, the clone designated as 3D8-1 was selected for large-scale antibody production, showing the highest specificity and reproducibility.

The clone producing the monoclonal antibody was then amplified, and aliquots of cells were prepared for cryopreservation.

The antibody’s classification was confirmed by analyzing its amino acid sequence. The analysis of constant heavy chain domains, specifically CH1, CH2, and CH3, provided definitive molecular evidence for a specific subclass, which is more reliable than migration patterns alone, as migration can be influenced by factors such as size and charge of molecules. Moreover, a Western blot analysis was performed using both reduced and non-reduced samples as follows. After large-scale expansion of the selected hybridoma clone, the antibody was purified from the culture supernatant through a standard downstream process based on protein G affinity chromatography, followed by buffer exchange into PBS and sterile filtration. The purification process (performed by a commercial service) yielded a highly homogeneous IgG preparation, as confirmed by SDS-PAGE analysis under reducing and non-reducing conditions. The total recovery of purified antibody was in the low milligram range, sufficient to support all the analytical and comparative assays reported in the study. Fifty microliters of the purified antibody were reduced by adding 5 µL of a mixture of 1 M dithiothreitol and 1 M ammonium bicarbonate [1:1], followed by incubation for 30 min at 37 °C. Subsequently, 1.5 µg of both reduced and non-reduced IgG-αGalomab were quantified by ELISA and then separated on a 10% (*w*/*v*) SDS-PAGE gel at 120 V and 30 mA for 1.5 h. The gel, which included 10 µL of Precision Plus Protein Dual Color Standard Marker (Bio-Rad, Hercules, CA, USA), was then stained with Coomassie Blue (Sigma-Aldrich/Merck). Stock solutions of IgG-αGalomab [385 ng/mL] were prepared and used for the validation tests.

### 2.4. Preparation of Bioprosthetic Commercial-like Tissue Samples

The animal tissues commonly used for manufacturing bioprostheses are primarily tendons, ligaments, and pericardium of bovine, porcine, and, less commonly, equine origin. To evaluate the effectiveness of αGal antigen recognition by IgG-αGalomab, bovine pericardial tissue was processed in a manner similar to the manufacturing method of bioprosthetic cardiac valves. The native bovine pericardium was collected from a certified abattoir (Inalca Spa, Castelvetro di Modena, Italy) and delivered to the laboratory under controlled conditions. After washing in sterile phosphate-buffered saline (PBS) at 4 °C, homogeneous pericardial samples were harvested from the anterior region of the heart. The specimens were inserted into 12 cm × 10 cm bespoke frames and treated with glutaraldehyde (GLU), according to the method previously described [21]: two steps of 24 hr in a buffered 0.625% (*v*/*v*) GLU solution, followed by storage in a buffered 0.2% (*v*/*v*) GLU solution.

### 2.5. Preparation of Red Meat Extract

Beef muscle was purchased from a local butcher. Two portions, approximately 100 g each, were obtained: one was cooked, and the other was kept raw for the subsequent experimental steps. Cooking was performed in boiling water and stopped when the internal temperature at the center of the meat reached 90 °C. Both raw and cooked meat samples were finely chopped and homogenized in 30 mL of PBS at pH 7.4, then centrifuged at 4500× *g* for 30 min at 4 °C. The resulting supernatants were filtered through a 0.8 μm membrane, lyophilized, and stored at −20 °C until further use. Protein content was determined using the QuantiPro BCA assay kit (Sigma-Aldrich/Merck).

### 2.6. ELISA Test

#### 2.6.1. Sensitivity Analysis

Polysorp Nunc-Immuno plates (Thermo Fisher Scientific, Waltham, MA, USA) were coated with 100 µL/well of different concentrations of αGal/HSA (Dextra Laboratories, NGP-3334): 1 µg/mL, 0.2 µg/mL, 0.04 µg/mL, 0.008 µg/mL, and 0.0016 µg/mL, and incubated for 1 h at 37 °C. Plates were then washed with 300 µL/w of PBS + 0.05% *v*/*v* Tween twice for 3 min and once for 5 min. Non-specific binding sites were blocked by incubation with 300 µL/w of 2% weight/volume HSA for 1 h at 37 °C. 100 µL/w of IgM-M86 at [1:20], [1:50], [1:150] and [1:450] and IgG-αGalomab at [1:1000], [1:10,000], [1:20,000], [1:40,000] and [1:60,000] were incubated on each coating concentration for 2 h at 37 °C. The dilution range of IgG-αGalomab was defined from the lowest detectable amount to the highest possible value. Concentrations above the upper limit would result in signal saturation, while concentrations below the detection threshold would be undetectable. All tested samples produced signals within the linear and quantifiable range of the ELISA. Plates were washed once again and then incubated with anti-mouse IgM [1:500] and IgG HRP conjugated antibodies (Jackson Immuno Research, West Grove, PA, USA) for 1 h at 37 °C. Finally, 100 mL of HRP substrate buffer was added to each well for 5 min, at RT, in the dark. The enzymatic reaction was stopped with 50 µL/well of 2M H_2_SO_4_. The plate absorbance was measured by a Multiskan Sky plate reader (Thermo Fisher Scientific) at 450 nm.

#### 2.6.2. Cross-Reactivity Assay

To evaluate the specific affinity of IgM-M86 and IgG-αGalomab antibodies against the αGal antigen, a cross-reaction assay was carried out using IgM-M86 and IgG at the concentrations of [1:50] and [1:20,000], respectively. Both antibodies were singularly incubated for 2 hr at 37 °C with raw and boiled red meat extract (RME and BME), galactobiose (GB), blood group B trisaccharide (BGB), lactose (LC), and αGal/HSA (each of the previous molecules was used at a concentration of 5 µg/mL) and 1% *w*/*v* BSA in constant agitation. 100 µL of each incubated sample was loaded onto Polysorp Nunc-Immuno plates, which were previously coated with 100 µL/well of αGal/HSA at 5 µg/mL (8 wells per single sample). The plates were then incubated for 1 h at 37 °C after blocking non-specific binding sites with 300 µL/well of 2% *w*/*v* HSA for 1 h at 37 °C. They were washed twice for 3 min and once for 5 min with PBS + 0.05% *v*/*v* Tween and then incubated with [1:500] of secondary anti-mouse IgM and IgG HRP conjugated antibodies for 1 h at 37 °C. Finally, 100 mL of HRP substrate buffer was added to each well for 5 min, at RT, in the dark. The enzymatic reaction was stopped with 50 µL/w of 2M H_2_SO_4_. The absorbance was measured by a Multiskan SkyHigh Plate Reader (Thermo Fisher Scientific) at 450 nm.

In the competitive ELISA, the antigen target (αGal) was first immobilized on the surface of the microplate wells at a concentration of 1 μg/mL. The detection antibodies (IgM-IgM-M86 and IgG-αGalomab) were pre-incubated with a solution containing competitors, i.e., bovine serum albumin, galactobiose, and lactose (Figure 1), which potentially bind to the antibodies and thereby inhibit their interaction with the plate-bound antigen. Only the fraction of the antibody that has not been bound by the competitors is able to bind to the antigen coated on the plate.

Data were reported as a relative measure of binding efficiency, calculated as (bound antibody/total antibody) × 100%.

### 2.7. αGal Detection via Absorbance in Bovine Pericardium

Polysorp Nunc-Immuno plates were coated with 100 µL/well of αGal/HSA at 5 µg/mL for 1 h at 37 °C. Plates were then washed with 300 µL/well of PBS + 0.05% *v*/*v* Tween twice for 3 min and once for 5 min. Nonspecific binding sites were saturated with 300 µL/w of 2% *w*/*v* HSA for 1 h at 37 °C. After washing, plates were incubated for 2 h at 37 °C with 100 µL/well of supernatant derived from the positive control (αGalomab/PBS) and the samples prepared in PBS. Plates were washed once again and then incubated with [1:500] of anti-mouse IgM and IgG HRP-conjugated antibodies for 1 h at 37 °C. Finally, 100 mL of HRP substrate buffer was added to each well for 5 min, at RT, in the dark. The enzymatic reaction was stopped with 50 µL/well of 2M H_2_SO_4_. The plate absorbance was measured by a Multiskan Sky plate reader at 450 nm.

The percentage reduction was calculated by comparing the maximum absorbance signal of the positive control with the absorbance values obtained from the tissue samples. This reduction corresponded to the relative amount of antibody that remained bound to the tissue during the incubation phase, reflecting the efficiency of the antigen–antibody interaction.

### 2.8. αGal Detection by Western Blot

1.5 µg of αGal/HSA (positive control) and HSA (negative control) were separated in a 10% *v*/*v* SDS/PAGE gel at 120 V and 30 mA for 1.5 h. After treatment with 10 µL of BLUeye Prestained Protein Ladder (Sigma Aldrich/Merck), one gel was stained with Coomassie, and one was electrophoretically transferred onto Amersham Protran nitrocellulose membrane (GE Healthcare, Chicago, IL, USA) at 300 V and 200 mA for 1 h in semi-dry conditions. Blocking of non-specific binding sites was achieved by incubation with 3% *w*/*v* HSA and 0.1% *v*/*v* Tween in tris-buffered saline for 2 h at RT. Nitrocellulose membrane strips (one for each single concentration of antibodies) were prepared and incubated overnight at 4 °C with decreasing dilutions of IgG-αGalomab (from [1:400,000] to [1:100,000]) or IgM-M86 antibody (from [1:50,000] to [1:500]). HSA-negative samples were used as controls and incubated with the highest concentration of both IgG-αGalomab [1:100,000] and M86 [1:500], in order to maximize sensitivity while detecting non-specific binding. Protein detection was achieved using HRP-conjugated anti-Mouse IgM or IgG (Thermo Fisher Scientific). The development of immunoreactivity was enhanced by incubating the membranes with SuperSignal West Pico chemiluminescence substrate for 3 min (Thermo Fisher Scientific). The target signal was visualized by exposing the photographic plate (Genesee Scientific, San Diego, CA, USA) for 12 min and developing it for a sufficient time to make the band of the sample, marked with the highest concentration of primary antibody, visible.

### 2.9. αGal Detection by Flow Cytometry

The specificity of IgG-αGalomab was evaluated in vitro using αGal epitope-expressing porcine primary kidney fibroblasts (PPKFs) (Cell Biologics Inc., Chicago, IL, USA) and αGal epitope-lacking human primary normal dermal fibroblasts (NDFa) (American Type Culture Collection, Manassas, VA, USA). PPKFs were seeded (1 × 10^4^ cells/cm^2^) on tissue culture dishes and cultured up to 70% confluence in complete fibroblast medium (Cell Biologics Inc.), under standard conditions (37 °C, 95% humidity, 5% CO_2_). In parallel, NDFa were maintained in DMEM-F12 (Sigma Aldrich/Merck) supplemented with 10% *v*/*v* FBS (Merck) and 1% *w*/*v* penicillin/streptomycin (Invitrogen, Darmstadt, Germany). For αGal analysis, PPKFs and NDFa were detached from culture dishes with EDTA/trypsin (Sigma Aldrich/Merck) and washed with 1% *w*/*v* BSA in PBS before staining with either IgG-αGalomab at [1:5], [1:10], and [1:25] dilution or IgM-M86 anti-αGal antibody at [1:25] dilution, in 100 µL of 1% *w*/*v* BSA buffer, for 45 min, at RT. The percentage of viable cells was evaluated using the trypan blue exclusion assay. Samples with 99% cell viability were used for flow cytometry analysis.

Samples were washed twice and then incubated in 200 µL of BSA-buffer with Alexa Fluor^®^ 488-conjugated AffinePure F(ab’)2 Fragment goat anti-mouse IgG+IgM (AF488-II Ab) (H+L; Jackson ImmunoResearch Laboratories, Inc., West Grove, PA, USA) at a [1:200] dilution, in 1% *w*/*v* BSA for 45 min at RT in the dark. Cells were washed twice and resuspended in 150 µL BSA buffer before loading on BD FACSCantoTM II system, (Becton Dickinson, San Jose, CA, USA) equipped with BD FACSDiva software v9.0. For each analysis, experiments were performed in triplicate and 1 × 10^4^ cells were acquired. Data were expressed as mean fluorescence intensity (MFI) ± standard error of the mean (SEM) and the percentage of positive cells.

### 2.10. αGalomab Application in Histological Studies

The liver, kidney, thymus, and skin of wild-type C57BL/6n mice were embedded in OCT compound (Tissue Tek; Sakura Finetek, Tokyo, Japan), cryo-cooled in liquid nitrogen, cut into 8-µm cryosections with Leica CM1520 microtome (Wetzlar, Germany), and transferred to Superfrost Plus Gold glasses (Thermo Fisher Scientific). To evaluate the general structure of tissue matrices, histological staining was performed using the Bio-Optica (Milan, Italy) “Rapid Frozen Sections H&E staining kit” according to the manufacturer’s protocol. Briefly, the glasses were washed in PBS for 10 min to dissolve the OCT, then immersed for 1 min in a hematoxylin solution. Samples were subsequently washed in tap water for 5 min, dipped 5 times in the development buffer, and rinsed again in tap water. Tissues were stained for 3 min with an eosin solution, followed by dehydration using 95% (*v*/*v*) ethanol (10 washes) and 100% (*v*/*v*) ethanol (1 wash). All specimens were mounted with non-aqueous mounting media. In parallel, the samples stained with IgG-αGalomab or IgM-M86 were analyzed using immunofluorescence and immunohistochemistry. All images were acquired by a DP74 Microscope Digital Camera (Olympus Corporation, Tokyo, Japan).

#### Immunofluorescence and Immunohistochemistry

For both immunofluorescence and immunohistochemistry, dilutions of 1:1000 and 1:100 were chosen as the optimal dilutions for IgG-αGalomab and IgM-M86, respectively.

The dilution of IgG-αGalomab [1:1000] and IgM-M86 [1:100] was determined empirically by performing preliminary titration experiments. A range of dilutions was tested to identify the conditions providing optimal signal-to-noise ratios and reproducibility.

For immunofluorescence, the tissue slices were washed in PBS for 10 min, then incubated with IgG-αGalomab or IgM-M86 antibodies and analyzed using a microscope ZL 200TFL (Z Lab S.r.l., Cerea (Verona), Italy). An additional staining with rabbit anti-human Collagen alpha-1(III) chain (COL3A1) antibody (AbCam, Cambridge, UK) and 4′,6-diamidino-2-phenylindole (DAPI; Merck) was performed. The detection of target antigens was carried out using a dilution of [1:100] for the following secondary antibodies: Rhodamine conjugated goat anti-mouse IgG (Sigma-Aldrich) for aGalomab; Alexa Fluor™ 555 conjugated goat anti-mouse IgM (Thermo Fischer) for IgM-M86; Fluorescein conjugated sheep anti-rabbit (Thermo Fisher) for COL3A1.

For immunohistochemistry, tissue slices were washed for 10 min in PBS to remove the OCT compound. The samples were then incubated with 2% *w*/*v* HSA for 15 min at RT to saturate non-specific binding sites. After washing in PBS for 2 min, the specimens were incubated with primary antibodies IgG-αGalomab [dilutions ranging from 1:5 to 1:1000] or IgM-M86 [dilutions ranging from 1:20 to 1:100] or IgM-M86 at the concentrations of [1:100] and [1:1000], respectively, for 1 h at 37 °C in a humidified chamber. The glasses were then washed three times in PBS for 5 min and incubated with 2% *w*/*v* HAS for 10 min. The incubation with secondary antibodies was carried out using [1:100] anti-mouse IgM or IgG HRP-conjugated antibodies for 1 h at 37 °C in a humidified chamber. The signal development was achieved by incubating the samples for 5 min in HRP substrate buffer under dark conditions. The enzymatic reaction was stopped by washing the glasses twice in deionized water for 5 min. Nuclear counterstaining was performed by immersing samples in hematoxylin (Bio-Optica) for 3 min.

### 2.11. Statistical Analysis

Data was analyzed using Microsoft Excel and Prism 7 for Windows (v7.03, GraphPad Software, Inc., San Diego, CA, USA). A two-sided unpaired T-test was used to assess significant differences between the activity of IgM-M86 and IgG-αGalomab, at the 0.95 confidence level. For flow cytometry, statistical significance was determined by one-way analysis of variance (ANOVA) followed by the Bonferroni post hoc test, comparing stained PPKFs to NDFa cells or AF488-II Ab-matched samples.

## 3. Results

### 3.1. Description of Experimental Data

#### 3.1.1. IgG-αGalomab Selection

**Figure 2:**
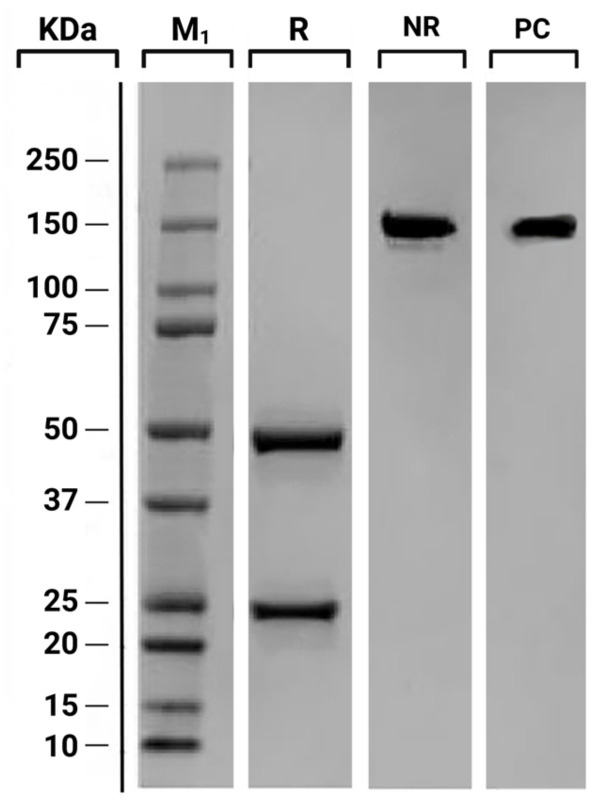
SDS-PAGE of purified mouse anti-αGal IgG1 (IgG-αGalomab) and commercial IgG1 (Positive Control, PC). Under reduced (R) conditions, IgG-αGalomab PC showed glycosylated heavy chains of approximately 50 kDa and light chains of around 25 kDa. Under non-reduced (NR) conditions, both IgG-αGalomab and the PC displayed a single band of about 150 kDa, consistent with the molecular weight of a complete IgG composed of two heavy and two light chains. M_1_: Protein Marker.

The supernatant from 10 selected hybridoma clones was used to test the sensitivity and specificity for αGal by ELISA assay. As reported in Table 1, clone 3D8-1 exhibited higher specificity against the epitope and a better signal-to-dilution ratio. Thus, it was selected for subsequent purification and testing by sequence analysis of constant regions of heavy chains (CH1–CH3) and SDS-PAGE under both reducing and non-reducing conditions. Sequence analysis showed a perfect match with the murine IgG1 isotype. As shown in Figure 2, when reduced, IgG-αGalomab antibody yielded glycosylated heavy chains of approximately 50 kDa and light chains of approximately 25 kDa. Under non-reducing conditions, a single band of approximately 150 kDa confirmed for IgG-αGalomab the regular molecular weight of a complete IgG, consisting of two heavy chains and two light chains [22].

#### 3.1.2. Sensitivity Assay

**Figure 3:**
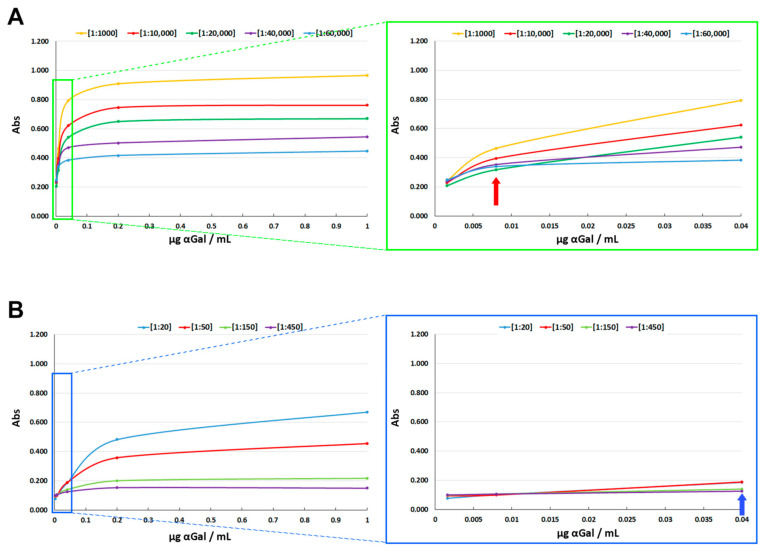
Detection sensitivity of IgG-αGalomab (**A**) and IgM-M86 (**B**). The analysis was performed by an ELISA test. At the maximum concentration of αGal [1 µg/mL], the absorbance (Abs) value of IgG-αGalomab [1:60,000] and IgM-M86 [1:50] was comparable. Notably, IgG-αGalomab from [1:1000] to [1:60,000] was functional in detecting αGal at a minimal concentration of 0.008 µg/mL ((**A**), red arrow). In contrast, IgM-M86 from [1:20] to [1:450] lost the ability to properly identify αGal with a concentration lower than 0.04 µg/mL ((**B**), blue arrow).

IgG-αGalomab, compared to IgM-M86, exhibited higher binding strength (*p* < 0.05) against the αGal antigen, as evidenced by its ability to detect the antigen at lower concentrations in ELISA tests. As reported in Figure 3, at the highest concentration used of αGal (1 µg/mL), IgG-αGalomab [1:60,000] (Figure 3A, blue line) revealed similar absorbance levels to those obtained with IgM-M86 [1:50] (Figure 3B, red line), thus exhibiting ~1200 times higher sensitivity to the antigen binding. For IgG-αGalomab and IgM-M86, the limit of detection (LOD) was 0.008 µg/mL and 0.04 µg/mL, respectively. Below these concentrations, a reliable recognition of the αGal antigen was not observed.

#### 3.1.3. Cross-Reactivity Assay

**Figure 4:**
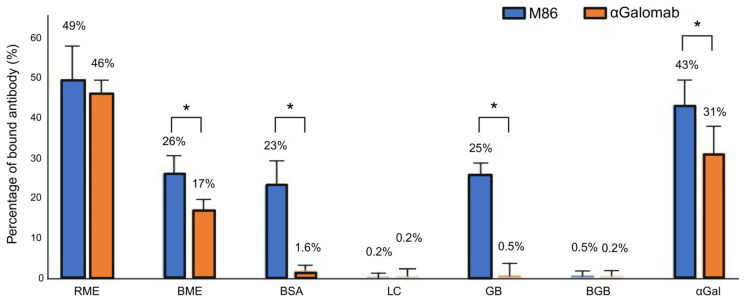
Cross-reactivity assay of IgG-αGalomab (orange color) and IgM-M86 (blue color) with raw meat extract (RME), boiled meat extract (BME), bovine serum albumin (BSA), lactose (LC), galactobiose (GB), blood group B (BGB), and αGal. The analysis was performed by a competitive ELISA test. Data were reported as a relative measure of binding efficiency, calculated as (bound antibody/total antibody) × 100% (*n* = 8 for each single test). * *p* < 0.05.

As shown in Figure 4, IgM-M86 exhibited a similar cross-reaction with both BSA and Galactobiose (GB) (23 ± 5.8% and 25 ± 4.3%, respectively). Conversely, IgG-αGalomab showed negligible binding to BSA (1.6 ± 1.2% reduction) and GB (0.5 ± 3%). Neither IgM-M86 nor IgG-αGalomab antibodies showed any interaction with lactose (LC) and blood group B trisaccharide (BGB). The IgM-M86 antibody demonstrated a similar reactivity to the αGal antigen and raw meat extract (43 ± 6.2% and 49 ± 8.7%, respectively; *p* > 0.05). In parallel, IgG-αGalomab demonstrated higher and statistically significant reactivity (*p* < 0.05) to raw meat extract than to αGal (46 ± 3.1% and 31 ± 6.6%, respectively).

As already reported in the literature, cooking meat is responsible for the inactivation of part of the antigens; however, both antibodies exhibited a sustained reactivity towards the BME samples. It is interesting to note that the non-specific reactivity towards BSA exhibited by the IgM-M86 antibody (23 ± 6%) is not statistically different (*p* > 0.05) from that obtained after incubation with the boiled meat extract (26 ± 4.1%, BME vs. BSA).

#### 3.1.4. αGal Detection in Bovine Pericardium

**Figure 5:**
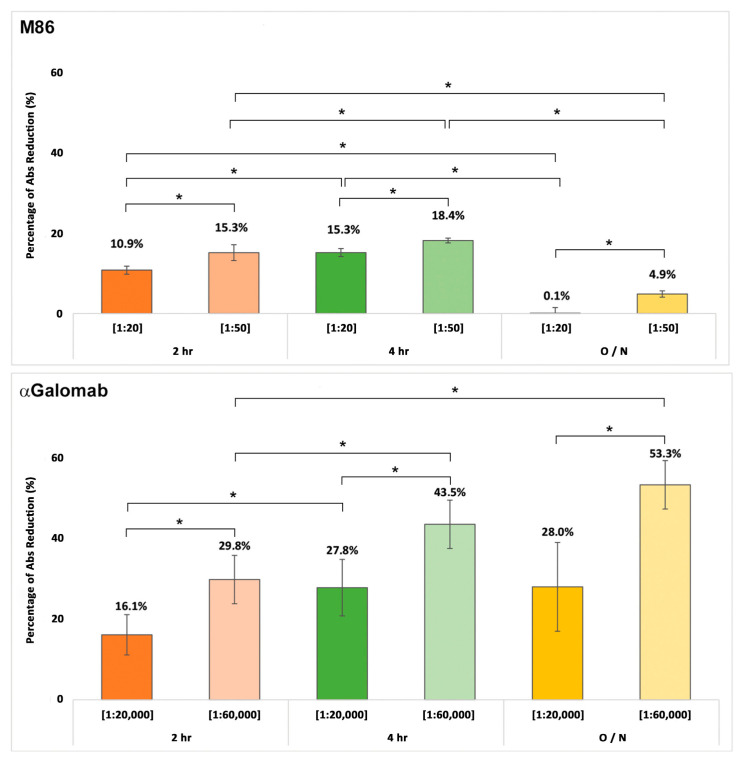
αGal detection in bovine pericardial tissue samples over time using IgG-αGalomab ([1:20,000], [1:60,000]) and IgM-M86 ([1:20], [1:50]). Data were expressed as a percentage of the measured absorbance (Abs) reduction. *n* = 8 replicas for every single dilution assessed; O/N = Overnight. * *p* < 0.05.

The results shown in Figure 5 are expressed as a percentage of signal reduction relative to the positive control. IgM-M86 [1:20] demonstrated an increasing signal reduction over time, while at dilution of [1:50], no significant differences in binding were observed during incubation. Globally, IgM-M86 showed more efficiency after 4 h of incubation at 37 °C. When incubated overnight, it drastically reduced its binding with the antigen, showing no signal at [1:20] and a negligible 7.8 ± 2.1% reduction at [1:50]. IgG-αGalomab, instead, used at concentrations 1000 times lower, showed a more sensitive and selective binding toward the epitope at all tested time points and increasing binding over time at [1:60,000] with a signal that decreased by 32.1 ± 5.9%, 48.6 ± 6.1%, and 59.9 ± 5.7% respectively at 2 h, 4 h and overnight (2 h vs. 4 h, *p* < 0.05; 4 h vs. overnight, *p* > 0.05). At [1:20,000], the anti-Gal IgG antibody showed a percentage of signal reduction lower than at [1:60,000] with no significant difference between 4 h and overnight incubation.

#### 3.1.5. Western Blot

**Figure 6:**
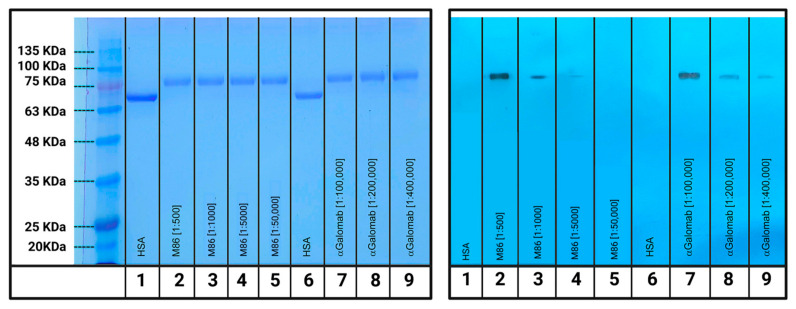
Electrophoresis (**on the left**) and western blot (**on the right**) analysis of IgG-αGalomab and IgM-M86 at different dilutions. Human serum albumin (HSA, column 1) was adopted as a negative control. Columns 1 and 6 are negative controls (HAS). Columns from 2 to 5 are marked with M86 antibody at [1:500] (2), [1:1000] (3), [1:5000] (4), and [1:50,000] (5). Columns from 7 to 9 are marked with IgG-αGalomab antibody at [1:100,000] (7), [1:200,000] (8), [1:400,000] (9).

As reported in Figure 6, IgM-M86 showed the ability to detect the αGal at the dilution of [1:500] and, to a lesser extent, at [1:1000] (columns 2 and 3, respectively). The signal became barely visible at [1:5000] and disappeared at [1:50,000] (columns 4 and 5, respectively). IgG-αGalomab, on the other hand, demonstrated a much higher sensitivity to αGal, binding the antigen at the dilutions of [1:100,000] and [1:200,000] (columns 7 and 8, respectively). Further lowering the concentration to [1:400,000], the signal became faintly visible (column 9). Neither antibody recognized the HSA negative control.

#### 3.1.6. Flow Cytometry

**Figure 7:**
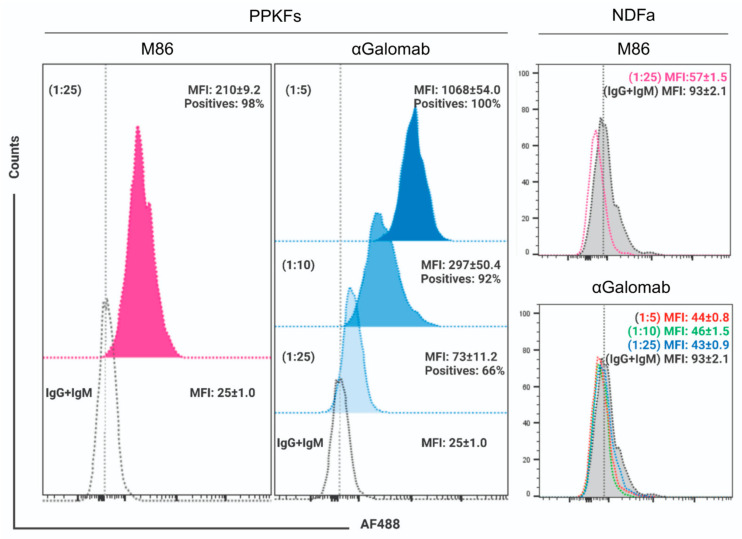
Flow cytometry analysis of αGal epitope in porcine primary kidney fibroblasts (PPKFs) and human primary normal dermal fibroblasts (NDFa) stained by indirect procedure with IgM-M86 antibody (fuchsia) at [1:25] dilution and IgG-αGalomab antibody (blue in PPKFs; red, green, and light blue for NDFa) at [1:25], [1:10], and [1:5] dilution. Samples treated with only AF488-conjugated secondary antibody were used as references (black dotted line). Data were reported as the mean percentage of positive cells and relative mean fluorescence intensity (MFI) ± SEM, calculated from *n* = 3 replicas of each sample for both cell populations. Histograms were created using FlowJo v.8.8.7 software (Becton Dickinson Biosciences, Franklin Lakes, NJ, USA) with normalization to mode.

The specificity of IgG-αGalomab was assessed using an in vitro model based on αGal epitope-expressing (porcine primary kidney fibroblasts, PPKFs) and non-expressing (human primary normal dermal fibroblasts, NDFa) cells. By FACS analysis, PPKFs compared to AF488-secondary antibody matched controls showed an almost homogenous expression (~100% positives; MFI: 210 ± 9.2) (*p* ≤ 0.01) of αGal after staining with IgM-M86 at [1:25] dilution (Figure 7). In parallel, a dose-dependent antigen binding wide spot (*p* ≤ 0.01) was observed in samples stained with IgG-αGalomab antibody at [1:5] (100% positives; MFI: 1068 ± 54.0), [1:10] (92% positives; MFI: 297 ± 50.4) and [1:25] dilution (66% positives; MFI: 73 ± 11.2). Conversely, no expression of αGal was measured in NDFa cells after staining with either IgM-M86 or IgG-αGalomab antibody (Figure 7).

#### 3.1.7. Microscopy

**Figure 8:**
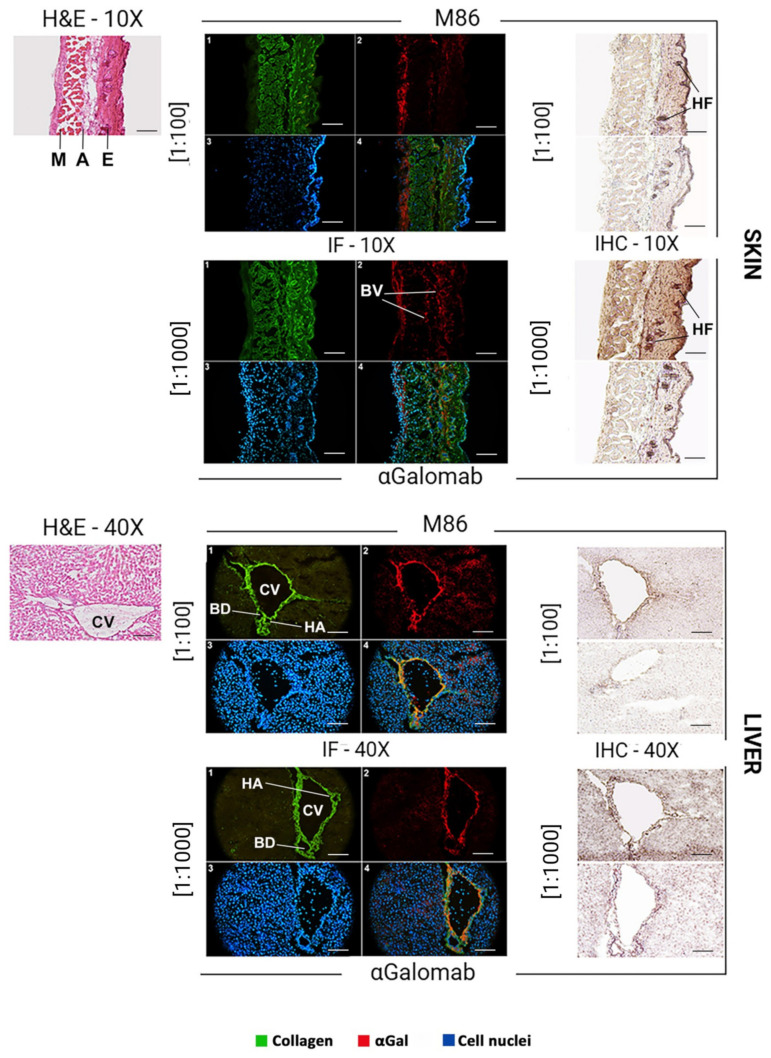
Brightfield microscopy (H&E, hematoxylin & eosin) (left), immunofluorescence (IF) (middle), and immunohistochemistry (IHC) (right) analysis to compare the staining quality of IgM-M86 (top) and IgG-αGalomab (bottom). Tissue regions highly expressing the αGal antigen have been identified. M: muscle; A: adipose tissue; E: epidermis; BV: blood vessel; HF: hair follicles; CV: central vein; BD: bile duct; HA: hepatic artery. (1) COL3A1 (green); (2) αGal (red); nuclei (blue); (4) merge. Scale bars: 100 μm (skin), 40 μm (liver).

**Figure 9:**
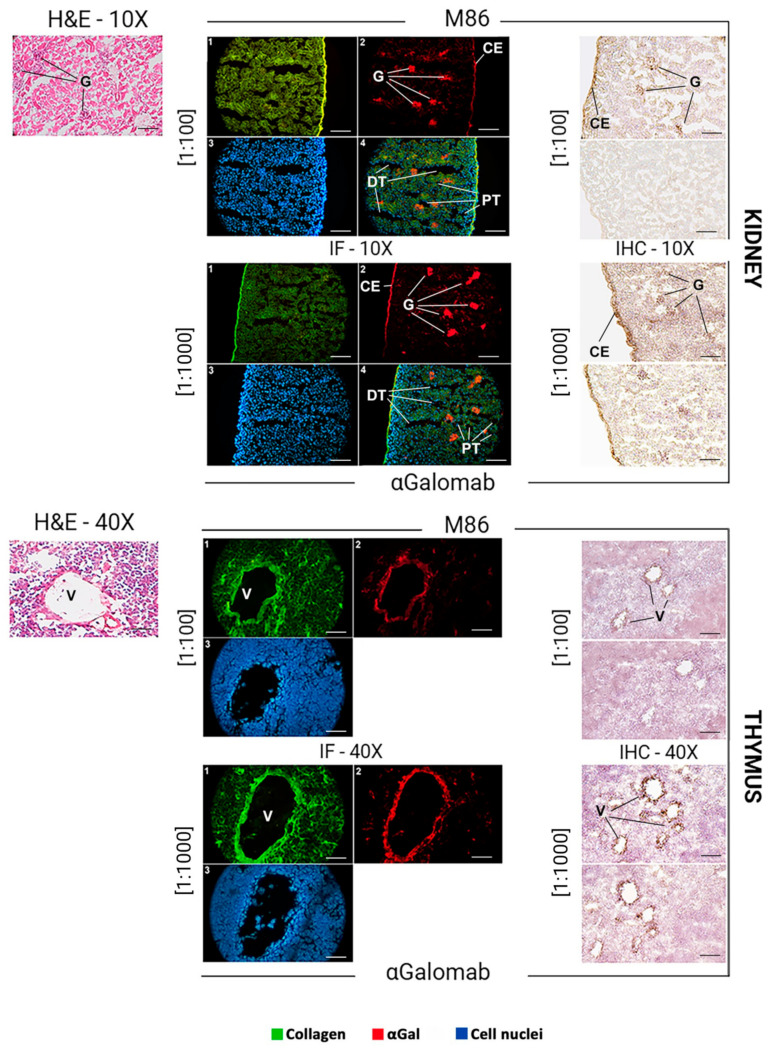
Detection of IgM-M86 (**top**) and IgG-αGalomab (**bottom**) via brightfield microscopy (H&E, hematoxylin & eosin) (**left**), immunofluorescence (IF) (**middle**), and immunohistochemistry (IHC) (**right)** in kidney and thymus mouse tissues. Tissue regions with high expression of the αGal antigen have been identified. G: renal glomerulus; CE: capsule endothelium; DT: distal tubules; PT: proximal tubules; V: venules. (1) COL3A1 (green); (2) αGal (red); nuclei (blue). Scale bars: 100 µm (kidney), 40 µm (thymus).

The αGal epitope is broadly expressed in murine tissues, with a distribution reflecting its association with glycoproteins and glycolipids on cell surfaces. In the mouse skin (Figure 8), αGal expression was mainly found in the endothelial cells of dermal blood vessels and hair follicles. In the liver (Figure 8), αGal was strongly expressed in endothelial cells, which line the hepatic sinusoids, as well as in the veins and arteries, and in epithelial cells. In the kidney (Figure 9), αGal was prominently detected in the vascular endothelium, particularly within glomerular and peritubular capillaries. Immunohistochemical staining also revealed its presence in the epithelial cells of the proximal and distal tubules, as well as in the endothelium of the capsule. In the thymus (Figure 9), αGal was identified in both cortical and medullary regions, with notable expression in thymic epithelial cells and vascular endothelium.

- Immunofluorescence

In all tissues analyzed (Figure 8, IF), to obtain a comparable signal intensity, IgM-M86 was used at a concentration 10-fold higher than IgG-αGalomab (IgM-M86 [1:100]; IgG-αGalomab [1:1000]). A higher screening sensitivity, especially in the skin and thymus, was observed in samples stained with the IgG antibody (IgG-αGalomab) compared to those incubated with IgM (IgM-M86).

- Immunohistochemistry

Both IgM-M86 and IgG-αGalomab antibodies successfully bound to the target antigen in all tissues examined, producing a visible signal and comparable background noise (Figure 8, IHC). However, IgG-αGalomab achieved an effective staining at both [1:100] and [1:1000] dilutions. In contrast, the IgM-M86 required a dilution of [1:100] (except for thymus) to reliably detect αGal, while showing low sensitivity when used at [1:1000], particularly in thymus, kidney, and liver.

## 4. Discussion

It is reported that the αGal (Galα1-3Galβ1-4GlcNAc-R) epitope has significant conformational flexibility, particularly at the α-(1→3) linkage [23], and the addition of even a single sugar unit strongly impacts its immunoreactivity [24]. The α1,3-galactosyltransferase enzyme (α1,3GT), which is encoded by the *Ggta1* gene, represents the main enzyme responsible for synthesizing the α-Gal on the cell surfaces of non-primate mammals. Located in the Golgi apparatus, it catalyzes the transfer of a uridine diphosphate-galactose (UDP-Gal) molecule to an N-acetyllactosamine (LacNAc) acceptor group, which is presented on proteins and lipids, forming an α(1,3)-glycosidic linkage [25]. The existence of another Gal-transferase was reported by Milland et al. [26], identifying isoglobo-tri-hexosylceramide synthase (iGb3s), able to bind the Galα1,3Gal disaccharide epitopes on glycosphingolipids (Lac) by the addition of galactose to lactosylceramide. Both the trisaccharide forms, LacNAc or Lac, are recognizable by the natural anti-αGal antibodies produced by the human immune system. In literature, the portion of the trisaccharide made up of Galα(1-4)GlcNAc is one of the most reactive parts of the antigen against antibodies [21]. Most likely, this part is more rigid and, therefore, could be more consistently recognized by the antibodies, becoming a key element in antigen recognition [27].

Currently, αGal is detectable using αGal-reactive lectins, antibodies purified by human sera, or a commercial monoclonal IgM M86 antibody. Lectins are a group of proteins of different origins that bind to cell surface sugars, typically forming hydrogen bonds and hydrophobic interactions with their ligands [28]. Due to this ability, their low cost, and ease of use, lectins are widely applied in the detection and study of polysaccharide structures, including αGal. However, lectins have been proven to have several limitations: Griffonia simplicifolia lectin IB4, peanut lectin [29], and Pseudomonas aeruginosa lectin I [30] recognize only the terminal galactose of the major carbohydrate xenoantigen, by hydrogen bonding to all its hydroxyl groups; the fungal galectin CGL2 interacts more strongly with the second galactose residue rather than the terminal galactose residue [31] and Clostridium difficile toxin A [32] and Marasmius oreades lectin (MOA) [33] engage in hydrogen bonding with several singular points throughout the carbohydrate. Moreover, the binding domain of lectins must interact with multiple αGal molecules to form a stable bond; as reported by Galili et al., these molecules are removed during the washing steps of the staining procedure [13]. Notwithstanding that the use of antibodies is reported to give more precise results in the detection of this xenoantigen [13,14,34], many investigations were still conducted using the lectin tool. Considering the literature to date, it appears that antibodies represent the most effective option for detecting αGal.

Several research groups obtain anti-αGal antibodies by purifying fresh human sera or sera treated for medical use (i.e., Octagam^®^, Beriglobulin^®^); this option is complex, expensive, and time-consuming, and it also requires a high level of expertise.

Given the lack of specificity of lectins and the complex purification process of human serum antibodies, a suitable practical choice is the M86 antibody, isolated in 1998 by Galili et al. [11]. It is a monoclonal mouse antibody belonging to the IgM class. IgM antibodies are typically secreted as soluble pentamers, offering ten binding sites for epitopes and thus exhibiting higher avidity for antigens compared to IgG immunoglobulins, which are only bivalently bound. Otherwise, IgM has the inherent characteristic of a lower binding affinity for the antigen and being more poly-reactive than other immunoglobulins [35,36]. Moreover, IgM antibodies are known to be more sensitive to pH balance, which, together with other components of the used buffer, such as proteins like BSA, can lead to their cleavage and impaired functionality, especially if they are of murine origin, as seen in IgM-M86 [37].

In this study, we produced an experimental IgG1 immunoglobulin, designated as IgG-αGalomab, with higher potency and specificity for the αGal antigen, as well as lower cross-reactivity, compared to the IgM-M86 antibody. Notably, our results demonstrated that IgG-αGalomab is highly selective against the most reactive and naturally available form of αGal, unlike the IgM-M86 clone, which also recognizes αGal1-3Gal disaccharide. Moreover, IgG-αGalomab also showed greater stability over time.

Based on the validation tests, IgG-αGalomab antibody appeared to be a suitable tool for αGal detection in various applications, including ELISA- or flow cytometry-based diagnostic assays, or microscopy analyses, such as immunofluorescence and immunohistochemistry assays, where, although used at concentrations 10 times lower than IgM-M86, its signal resulted sharper and more distinct than that of IgM-M86. The instability of the IgM-M86 antibody compared to other antibodies stems from the general properties of IgM and its potential for inconsistent performance. Factors such as storage conditions, its pentameric structure, and its sensitivity to degradation over time can contribute to its instability, although it has high avidity due to its multiple binding sites. This can result in varying results, as highlighted in the comparison study with IgG-αGalomab. Belonging to the IgG family, which is typically considered the better choice for most IHC/IF applications, αGalomab, compared to M86, exhibits a lower molecular mass, thereby allowing for improved performance and signal resolution, as well as a significant reduction in non-specific binding of secondary antibodies or cross-reactivity with other antibodies. The stronger reactivity of IgG-αGalomab to raw meat extract compared to purified αGal was likely dependent on a different epitope density. In literature, bovine kidney or liver is reported to contain approximately 10^11^–10^12^ αGal epitopes/µg of total proteins. We speculated that the supernatant from the tissue extraction process could contain a comparable quantity of αGal epitopes, and thus, the raw meat extract could be characterized by an intrinsically higher epitope density, which led IgG to be more reactive to the extract compared to the purified αGal solution. No limitations were identified beyond the specific scope of the comparative analysis with M86 for αGalomab.

Recently, regulatory agencies in the USA. (i.e., the FDA) and in Europe (i.e., the European Commission) have not explicitly set universal guidelines to detect αGal; instead, their focus is on characterizing the αGal oligosaccharide within biological products (foods, drugs, bioprostheses, medical devices) and monitoring αGal-related food allergies (Alpha-gal Syndrome) in humans. This will enable greater food safety and transparency, detailed food labeling (allowing consumers with sensitivity to avoid foods that could trigger allergic reactions, similar to existing labeling for other food allergies, such as gluten or peanuts), and increased consumer awareness. People allergic to αGal will have greater control over their diet, which can significantly reduce the risk of severe allergic reactions, such as anaphylaxis. This is expected to enhance patients’ quality of life and reduce healthcare costs associated with emergency interventions.

The ability to accurately measure αGal in drugs and bioprosthetic medical devices will increase safety and innovation in their manufacturing. In fact, although the recent FDA approval of αGal knockout pigs (GalSafe) represents a significant advancement [12], the high costs of their production and maintenance, along with the limited yield of organs and tissues per animal, pose substantial barriers. These factors are likely to drive up the final cost of bioprosthetic devices, thereby reducing their accessibility and market competitiveness. Consequently, many companies engaged in the production of bioprosthetic devices are actively exploring alternative technologies to overcome the challenge of biocompatibility. In this context, the development and implementation of an effective and reliable method for verifying antigen removal or inactivation represents a critical step toward ensuring the safety and efficacy of these devices. Preventing adverse reactions by measuring αGal levels in animal-derived materials used for drug or bioprosthetic device production enables physicians to avoid administering αGal-containing products to patients who are sensitized, thereby reducing the risk of rejection and immune responses. Additionally, preoperative testing can further enhance compatibility among these medical products and individuals who are sensitized to them. Furthermore, access to data on αGal levels will drive research into the creation of biocompatible materials free of αGal, such as synthetic or engineered prostheses. This will stimulate innovation and increase the range of safe options for patients.

## 5. Conclusions

In precision medicine, there is an increasing demand for more effective tools of αGal detection in animal-derived materials. The results presented in this study demonstrate that IgG-αGalomab is a promising candidate for an advanced αGal detection tool, adaptable to multiple analytical methods and substrate types, and exhibiting sensitivity and specificity that surpass current standard assays. A label-free binding assay, such as isothermal titration calorimetry (ITC), will provide future insight into the functional analysis of the IgG1 antibody compared to M86, to define its complete structure-activity-thermodynamics profile.

## Figures and Tables

**Table 1:** IgG-αGalomab titer in clone supernatants. The analysis was performed by direct ELISA, using plates coated with 5 µg/mL of αGal/HSA (A) or HSA (B).

Cell Lines	Supernatant Dilution						Negative Control	Titer	Coating
	[1:10]	[1:30]	[1:90]	[1:270]	[1:810]	[1:2430]			
1G11-1	3.122	2.992	2.871	2.530	2.192	1.321	0.080	>1:2, 430	A
1G11-1	0.116	0.109	0.101	0.098	0.103	0.096	0.090	<1:10	B
1G11-2	2.889	2.875	2.705	2.585	2.083	1.346	0.080	>1:2, 430	A
1G11-2	0.109	0.117	0.095	0.102	0.096	0.099	0.090	<1:10	B
2B12-1	2.908	2.773	2.552	1.994	1.273	0.651	0.080	>1:2, 430	A
2B12-1	0.110	0.104	0.095	0.092	0.087	0.105	0.090	<1:10	B
2B12-2	2.692	2.787	2.500	2.095	1.255	0.642	0.080	>1:2, 430	A
2B12-2	0.110	0.104	0.086	0.097	0.092	0.086	0.090	<1:10	B
3D8-1	2.922	2.755	2.288	1.556	0.865	0.426	0.080	>1:2, 430	A
3D8-1	0.107	0.092	0.102	0.092	0.085	0.087	0.090	<1:10	B
3D8-2	2.910	2.896	2.443	1.869	1.065	0.558	0.080	>1:2, 430	A
3D8-2	0.114	0.114	0.097	0.097	0.092	0.105	0.090	<1:10	B
16F10-1	3.013	2.947	2.739	2.465	2.003	1.346	0.080	>1:2, 430	A
16F10-1	0.092	0.093	0.091	0.097	0.084	0.110	0.090	<1:10	B
16F10-2	2.978	2.921	2.565	2.442	1.941	1.306	0.080	>1:2, 430	A
16F10-2	0.094	0.088	0.079	0.105	0.104	0.112	0.090	<1:10	B
20D12-1	2.835	2.874	2.686	2.418	1.680	1.145	0.080	>1:2, 430	A
20D12-1	0.116	0.102	0.109	0.101	0.095	0.104	0.090	<1:10	B
20D12-2	2.792	2.802	2.505	1.960	1.280	0.638	0.080	>1:2, 430	A
20D12-2	0.096	0.087	0.088	0.088	0.080	0.100	0.090	<1:10	B

## Data Availability

The original contributions presented in this study are included in the article. Further inquiries can be directed to the corresponding author(s).

## Abbreviations

HSA/Human Serum Albumin; BSA/Bovine Serum Albumin; GLU/Glutaraldehyde; RME/Raw Meat Extract; BME/Boiled Meat Extract; GB/Galactobiose; BGB/Blood Group B; LC/Lactose; HRP/Horseradish Peroxidase; PPKFs/Porcine Primary Kidney Fibroblasts; NDFs/Normal Dermal Fibroblasts; OCT/Optimal Cutting Temperature.

## References

[1] Galili U., Shohet S.B., Kobrin E., Stults C.L., Macher B.A. (1988). Man, apes, and Old World monkeys differ from other mammals in the expression of alpha-galactosyl epitopes on nucleated cells. J. Biol. Chem..

[2] Hamadeh R.M., Galili U., Zhou P., Griffiss J.M. (1995). Anti-alpha-galactosyl immunoglobulin A (IgA), IgG, and IgM in human secretions. Clin. Diagn. Lab. Immunol..

[3] Montassier E., Al-Ghalith G.A., Mathé C., Le Bastard Q., Douillard V., Garnier A., Guimon R., Raimondeau B., Touchefeu Y., Duchalais E. (2020). Distribution of Bacterial α1,3-Galactosyltransferase Genes in the Human Gut Microbiome. Front. Immunol..

[4] Perusko M., Grundström J., Eldh M., Hamsten C., Apostolovic D., van Hage M. (2024). The α-Gal epitope—The cause of a global allergic disease. Front. Immunol..

[5] Calafiore A.M., Haverich A., Gaudino M., Di Mauro M., Fattouch K., Prapas S., Zilla P. (2022). Immunoreaction to xenogenic tissue in cardiac surgery: Alpha-Gal and beyond. Eur. J. Cardiothorac. Surg..

[6] Senage T., Paul A., Le Tourneau T., Fellah-Hebia I., Vadori M., Bashir S., Galiñanes M., Bottio T., Gerosa G., Evangelista A. (2022). The role of antibody responses against glycans in bioprosthetic heart valve calcification and deterioration. Nat. Med..

[7] Veraar C., Koschutnik M., Nitsche C., Laggner M., Polak D., Bohle B., Mangold A., Moser B., Mascherbauer J., Ankersmit H.J. (2021). Inflammatory immune response in recipients of transcatheter aortic valves. JTCVS Open.

[8] Bozso S.J., El-Andari R., Al-Adra D., Moon M.C., Freed D.H., Nagendran J., Nagendran J. (2021). A review of the immune response stimulated by xenogenic tissue heart valves. Scand. J. Immunol..

[9] Kostyunin A.E., Yuzhalin A.E., Rezvova M.A., Ovcharenko E.A., Glushkova T.V., Kutikhin A.G. (2020). Degeneration of Bioprosthetic Heart Valves: Update 2020. J. Am. Heart Assoc..

[10] Swiontek K., Morisset M., Codreanu-Morel F., Fischer J., Mehlich J., Darsow U., Petitpain N., Biedermann T., Ollert M., Eberlein B. (2019). Drugs of porcine origin-A risk for patients with α-gal syndrome?. J. Allergy Clin. Immunol. Pract..

[11] Copic D., Bormann D., Direder M., Ankersmit H.J. (2022). Alpha-Gal-specific humoral immune response and reported clinical consequence for cardiac valve replacement in patients below 65 years: Moving beyond conjecture. Eur. J. Cardiothorac. Surg..

[12] U.S. Food and Drug Administration https://www.prnewswire.com/news-releases/fda-approves-first-of-its-kind-intentional-genomic-alteration-in-line-of-domestic-pigs-for-both-human-food-potential-therapeutic-uses-301192244.html.

[13] Galili U., LaTemple D.C., Radic M.Z. (1998). A sensitive assay for measuring alpha-Gal epitope expression on cells by a monoclonal anti-Gal antibody. Transplantation.

[14] Kuravi K.V., Sorrells L.T., Nellis J.R., Rahman F., Walters A.H., Matheny R.G., Choudhary S.K., Ayares D.L., Commins S.P., Bianchi J.R. (2022). Allergic response to medical products in patients with alpha-gal syndrome. J. Thorac. Cardiovasc. Surg..

[15] Cunningham S., Starr E., Shaw I., Glavin J., Kane M., Joshi L. (2013). Development of a convenient competitive ELISA for the detection of the free and protein-bound nonhuman galactosyl-α-(1,3)-galactose epitope based on highly specific chicken single-chain antibody variable-region fragments. Anal. Chem..

[16] Kreft L., Schepers A., Hils M., Swiontek K., Flatley A., Janowski R., Mirzaei M.K., Dittmar M., Chakrapani N., Desai M.S. (2022). A novel monoclonal IgG1 antibody specific for Galactose-alpha-1,3-galactose questions alpha-Gal epitope expression by bacteria. Front. Immunol..

[17] Naso F., Stefanelli U., Buratto E., Lazzari G., Perota A., Galli C., Gandaglia A. (2017). Alpha-Gal Inactivated Heart Valve Bioprostheses Exhibit an Anti-Calcification Propensity Similar to Knockout Tissues. Tissue Eng. Part A.

[18] Naso F., Colli A., Zilla P., Calafiore A.M., Lotan C., Padalino M.A., Sturaro G., Gandaglia A., Spina M. (2023). Correlations between the alpha-Gal antigen, antibody response and calcification of cardiac valve bioprostheses: Experimental evidence obtained using an alpha-Gal knockout mouse animal model. Front. Immunol..

[19] Naso F., Gandaglia A., Sturaro G., Galli C., Melder R.J. (2024). The α-Gal KO Mouse Animal Model is a Reliable and Predictive Tool for the Immune-Mediated Calcification Assessment of Heart Valve Bioprostheses. Front. Biosci..

[20] Köhler G., Milstein C. (1975). Continuous cultures of fused cells secreting antibody of predefined specificity. Nature.

[21] Stacchino C., Bona G., Bonetti F., Rinaldi S., Della Ciana L., Grignani A. (1998). Detoxification process for glutaraldehyde treated bovine pericardium: Biological, chemical and mechanical characterization. J. Heart Valve Dis..

[22] Kirley T.L., Norman A.B. (2018). Unfolding of IgG domains detected by non-reducing SDS-PAGE. Biochem. Biophys. Res. Commun..

[23] Agostino M., Sandrin M.S., Thompson P.E., Yuriev E., Ramsland P.A. (2009). In silico analysis of antibody-carbohydrate interactions and its application to xenoreactive antibodies. Mol. Immunol..

[24] Santra G., Pantazis D.A. (2025). Conformational Profile of Galactose-α-1,3-Galactose (α-Gal) and Structural Basis of Its Immunological Response. Chemistry.

[25] Strahan K.M., Gu F., Preece A.F., Gustavsson I., Andersson L., Gustafsson K. (1995). cDNA sequence and chromosome localization of pig alpha 1,3 galactosyltransferase. Immunogenetics.

[26] Milland J., Christiansen D., Lazarus B.D., Taylor S.G., Xing P.X., Sandrin M.S. (2006). The molecular basis for galalpha(1,3)gal expression in animals with a deletion of the alpha1,3galactosyltransferase gene. J. Immunol..

[27] Anraku K., Sato S., Jacob N.T., Eubanks L.M., Ellis B.A., Janda K.D. (2017). The design and synthesis of an α-Gal trisaccharide epitope that provides a highly specific anti-Gal immune response. Org. Biomol. Chem..

[28] Osterne V.J., De Sloover G., Van Damme E.J. (2024). Revisiting legume lectins: Structural organization and carbohydrate-binding properties. Carbohydr. Res..

[29] Natchiar S.K., Srinivas O., Mitra N., Surolia A., Jayaraman N., Vijayan M. (2006). Structural studies on peanut lectin complexed with disaccharides involving different linkages: Further insights into the structure and interactions of the lectin. Acta Crystallogr. D Biol. Crystallogr..

[30] Blanchard B., Nurisso A., Hollville E., Tétaud C., Wiels J., Pokorná M., Wimmerová M., Varrot A., Imberty A. (2008). Structural basis of the preferential binding for globo-series glycosphingolipids displayed by Pseudomonas aeruginosa lectin I. J. Mol. Biol..

[31] Walser P.J., Haebel P.W., Künzler M., Sargent D., Kües U., Aebi M., Ban N. (2004). Structure and functional analysis of the fungal galectin CGL2. Structure.

[32] Greco A., Ho J.G.S., Lin S.-J., Palcic M.M., Rupnik M., Ng K.K.-S. (2006). Carbohydrate recognition by Clostridium difficile toxin A. Nat. Struct. Mol. Biol..

[33] Grahn E., Askarieh G., Holmner Å., Tateno H., Winter H.C., Goldstein I.J., Krengel U. (2007). Crystal structure of the Marasmius oreades mushroom lectin in complex with a xenotransplantation epitope. J. Mol. Biol..

[34] Naso F., Gandaglia A., Bottio T., Tarzia V., Nottle M.B., D’Apice A.J.F., Cowan P.J., Cozzi E., Galli C., Lagutina I. (2013). First quantification of alpha-Gal epitope in current glutaraldehyde-fixed heart valve bioprostheses. Xenotransplantation.

[35] Keyt B.A., Baliga R., Sinclair A.M., Carroll S.F., Peterson M.S. (2020). Structure, Function, and Therapeutic Use of IgM Antibodies. Antibodies.

[36] Schroeder H.W., Cavacini L. (2010). Structure and function of immunoglobulins. J. Allergy Clin. Immunol..

[37] Klaus T., Stalińska K., Czaplicki D., Mak P., Skupien-Rabian B., Kedracka-Krok S., Wiatrowska K., Bzowska M., Machula M., Bereta J. (2018). Mouse Antibody of IgM Class is Prone to Non-Enzymatic Cleavage between CH1 and CH2 Domains. Sci. Rep..

